# Adaptive Whisking in Mice

**DOI:** 10.3389/fnsys.2021.813311

**Published:** 2022-01-27

**Authors:** Shubhodeep Chakrabarti, Jithin Nambiar, Cornelius Schwarz

**Affiliations:** Systems Neurophysiology, Werner Reichardt Center for Integrative Neuroscience, Hertie-Institute for Clinical Brain Research, University of Tübingen, Tübingen, Germany

**Keywords:** primary motor cortex, head-fixed, whisker movement, lesion, mouse

## Abstract

Rodents generate rhythmic whisking movements to explore their environment. Whisking trajectories, for one, appear as a fixed pattern of whisk cycles at 5–10 Hz driven by a brain stem central pattern generator. In contrast, whisking behavior is thought to be versatile and adaptable to behavioral goals. To begin to systematically investigate such behavioral adaptation, we established a whisking task, in which mice altered the trajectories of whisking in a goal-oriented fashion to gain rewards. Mice were trained to set the whisker to a defined starting position and generate a protraction movement across a virtual target (no touch-related tactile feedback). By ramping up target distance based on reward history, we observed that mice are able to generate highly specific whisking patterns suited to keep reward probability constant. On a sensorimotor level, the behavioral adaptation was realized by adjusting whisker kinematics: more distant locations were targeted using higher velocities (i.e., pointing to longer force generation), rather than by generating higher acceleration (i.e., pointing to stronger forces). We tested the suitability of the paradigm of tracking subtle alteration in whisking motor commands using small lesions in the rhythmic whisking subfield (RW) of the whisking-related primary motor cortex. Small contralateral RW lesions generated the deterioration of whisking kinematics with a latency of 12 days post-lesion, a change that was readily discriminated from changes in the behavioral adaptation by the paradigm.

## Introduction

Voluntary movements are generated by neuronal systems that identify goals and assure adequate behavior in varying contexts (executive signals), devise adequate trajectories (kinematics), and translate them into low-level motor commands driving the muscles (dynamics). From a neuroanatomy perspective, the motor system is hierarchically organized into higher-order prefrontal/premotor cortical areas, primary motor cortex, pattern generators in the brain stem and spinal cord, and motor neurons. The correspondence of function and neuronal substrate is far from settled and is an active field of research in the motor system of primates (Schieber, 2001; Graziano, 2006; Shenoy et al., 2013) and rodents (Chakrabarti and Schwarz, 2015; Guo et al., 2015; Kawai et al., 2015). To extract functions on specific levels of the neuronal organization of motor function, precise behavioral tasks are needed, which are able to monitor and test relevant behavioral variables on the different levels mentioned. In primates, the center-out reaching task and its variants provide a rich toolbox to study goal-directed motor behavior on all levels of organization (Georgopoulos et al., 1982; Kalaska et al., 1989; Moran and Schwartz, 1999; Scott, 2000; Churchland et al., 2012). In the whisker system, which is the best-studied rodent sensorimotor system, a comparable behavioral standard is missing. Repetitive reaching tasks are available (Hentschke et al., 2006; Huber et al., 2012), as well as tasks geared toward studying active discrimination (Chen et al., 2013; Georgieva et al., 2014; Banerjee et al., 2020), or selective detection (Aruljothi et al., 2020). This study aims to close a gap to study the middle ground between higher-order executive signals and generation of low-level motor command by bringing a goal-oriented adaptation of movement trajectories under experimental control. We introduced a novel behavioral task that motivates mice to adaptively change their whisking motor command by systematically changing the spatial geometric context of the task. Mice-generated whisker reaches to hit a virtual target (no sensory feedback) that would change its location systematically during a behavioral session. We observed that mice, based solely on available reward information, were able to fine-tune their whisker trajectories in a goal-oriented manner according to these changes. We tested the suitability of the task to probe the neuronal underpinning of motor adaptability by testing whisker reaching adaptations after the application of small lesions to contralateral rhythmic whisking (RW), a subarea of the whisker-related primary motor cortex (wM1), that has been shown to give rise to protraction and rhythmic whisker movement on microstimulation (Haiss and Schwarz, 2005; Cramer and Keller, 2006; Ferezou et al., 2007). After applying RW lesions, whisker adaptation stayed unaffected for about 2 weeks (12 days). Later, the test showed increasing deterioration of reach adaptation.

## Materials and Methods

### Head-Post Implantation

Three male CS57/Bl6 mice were used in this study. All experimental and surgical procedures were performed in accordance with the policies on the use of animals in neuroscience research of the Society for Neuroscience and German Law. Anesthesia was initialized with isoflurane (2%). After making sure that the responses to tail pinches were suppressed, the anesthesia was continued by an intraperitoneal (i.p.) injection of 3K (fentanyl 0.5 mg/kg, midazolam 12.5 mg/kg, and fluanisone 25 mg/kg, i.p.) and upheld by refresher injections of 33% of the initial dose every 50 min. The skin covering the dorsal skull was shaved, and the mice were transferred to a stereotaxic frame in the absence of response to tail pinches, where the rectal temperature of the animal was controlled automatically by a feedback circuit composed of a rectal probe and a heating pad and set to 35°C. Following skin excision and careful removal of underlying connective tissue, mice were fitted with a dental cement head-post positioned so as not to invade the skull area rostral to bregma where the location of the RW module (coordinates rostral 3, lateral 2 mm from bregma) was marked using an ink marker. A head-post (M1 stainless steel screw, head-down) was embedded within the dental cement. The wound was attached to the head post and carefully sutured. The still anesthetized mice were first given the painkiller carprofen (5 mg/kg) followed by 3K antidote (naloxone 1.20 mg/kg, flumazenil 0.50 mg/kg, and atipamezole 2.50 mg/kg, i.p.), on which anesthesia was reversed within a few minutes. Everyday post-surgery care included the continuation of carprofen injection (two times a day for minimally 3 days) and, if needed, the administration of warmth and electrolytes. After the behavioral training and experiments, the animals were killed under deep narcosis (pentobarbital 100 mg/kg) by the intracardial perfusion of formaldehyde.

### Behavioral Training

**FIGURE 1:**
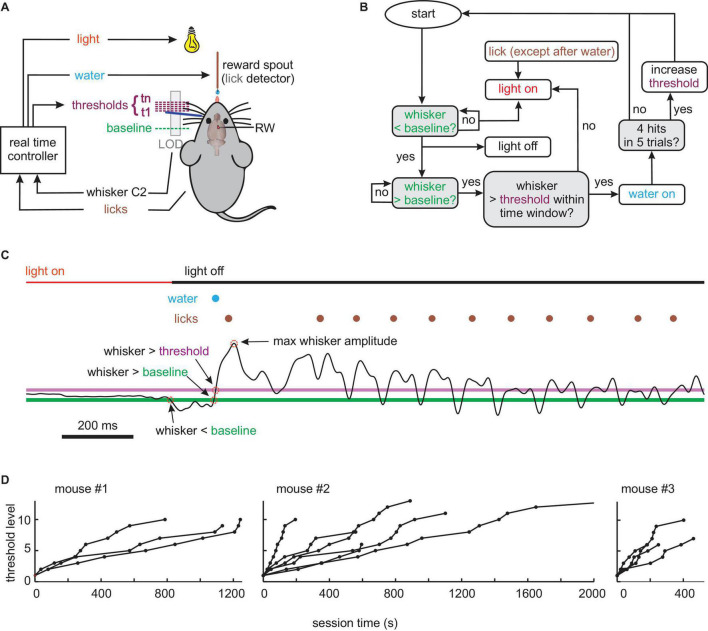
Behavioral **(A)** setup. The movement of whisker C2 (dark blue) of a head-fixed mouse is monitored in real-time by a laser optical device (LOD). The task of the mouse is to bring its whisker in starting position behind a baseline. A trial is started by protracting C2 across a baseline and successfully concluded (rewarded by a drop of water) by crossing a variable threshold. Light OFF signals that a trial can be started. **(B)** Flowchart of experimental control. Colors match those in panels **(A,C)**. Decision points are marked with gray boxes. **(C)** Example of a successful trial, i.e., going to a start position behind the baseline (green, first red circle) turning the house light OFF; moving forward across baseline (second red circle) and threshold (magenta, third red circle) within a preset time, reward delivery (blue), and licking (brown). Whisker trajectory is the thin black line (whisk amplitude fourth red circle). Colors match those in panels **(A,B)**. **(D)** Progression of threshold levels (threshold increment per level: 0.93 mm) across session time in all pre-lesion sessions of the three mice.

Habituation of mice to the experimenter, setup, and head-fixation and water restriction were begun earliest a week after head-cap implantation. The procedure exactly followed a published one and ended once the animals were successfully habituated and comfortable with head-fixation (Schwarz et al., 2010). Before the training of the whisker movement started, all but one whisker (C2) on the left side of the snout were trimmed to 2 mm in length; C2 was left a little longer, and whiskers on the right side of the snout were left intact. For behavioral sessions (Figure 1A), the animals were head-fixed, and a thin black polyimide tube (length 2 cm, weight 0.4 g) was fitted onto the C2 whisker for the detection of the whisker trajectories. The training took place in a vented and dark cubicle. Whisking was monitored optoelectronically using a laser optical device consisting of a 2D laser curtain positioned to illuminate a 1D camera chip (MX series, Metralight Inc., San Mateo, CA, United States). The movement of the sheathed whisker would interrupt the 2D laser and throw a shadow on the camera chip, which in turn was converted into a voltage readout (length of the chip 2.8 cm; spatial resolution 0.4375 μm; frame rate 2.5 kHz). The whisker position, therefore, was accessible in real-time during the movement. The laser curtain was positioned with a vertical beam at a distance of 0.5 cm lateral to the snout of the mouse, so that the rostrocaudal whisker movement component was recorded. The position of the 1D camera chip was adjusted for each mouse, such that the resting position of the C2 whisker was within the caudal third of the spatial extension of the chip, and the large protraction movements of typically up to around 50° could be monitored without the whisker leaving the range of the chip.

**FIGURE 2:**
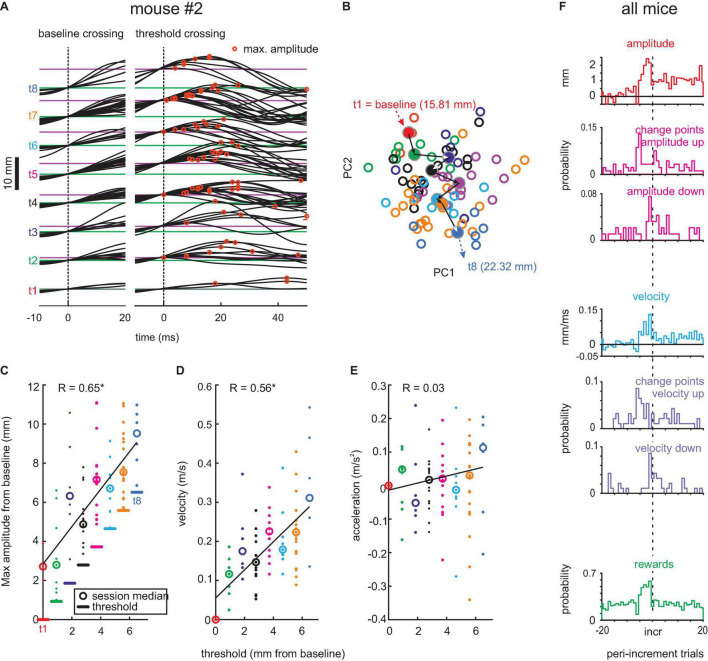
Adaptive whisking. **(A)** Example session. Whisker trajectories triggered by baseline crossing (green, left) and threshold crossings (magenta, right). Eight threshold levels (t1–t8) were reached in this session. Red circles mark maximum whisker position. **(B)** Plot of coefficient of first and second principal components (PC1 and PC2) of waveforms generated with each threshold (colors match those in the other panels). Circles: single trials. *Dots*: Center of mass for each threshold. *Arrows*: Mean trajectory across the 8 thresholds. Thresholds in mm, as given here, translate to degrees as follows $\left[2,4,6\right]\,\text{mm}\,\hat{=}\,\left[22,39,50\right]\,\text{deg}$. **(C)** Amplitudes reached with each threshold. Linear regression in black. **(D)** Same as in panel **(C)** for maximum whisker velocity. **(E)** Same for maximum acceleration. **(F)** Population data. Histograms of maximal amplitude (red), maximal velocity (blue), and rewards (green) on the peri-increment trials (zero is the increment trial; bin size = 1 trial). Histograms of change points taken from the trial series of maximal amplitude (magenta) and maximal velocity (violet). All change points exceeding the confidence level of 0.7 are shown. Maximal amplitude and velocity (red and blue histograms) are plotted relative to the mean value observed in trials -20 to -10 (indicated by zero) to emphasize the dynamics of these kinematic parameters around the increment trial.

After fixing the whisker tracking, the training commenced with one session, in which the water spout emitted a drop of water every 1 s, to train licking. From there on, water was made contingent to whisking. The training procedure (controlled, and monitored in real-time using SIMULINK, Natick, MA, United States) is shown in Figure 1B. For each session, a “baseline” was chosen at a location rostral to resting position. A “threshold” was then automatically set by the experiment controlling software coinciding with the baseline in the beginning of the session. Depending on the performance of the animal, the threshold dynamically changed its location during the session. The house light was controlled by the behavior of the animal in real-time and indicated whether a trial was enabled or disabled. Light OFF, which is the more comfortable situation for mice, indicated that the trial was ready to be started. The light was set to OFF if the whisker was located behind the baseline, and licks were withheld. Light ON, which indicated that the trial was disabled, was set when the whisker position was rostral to the baseline, licks occurred, or the sequential crossing of baseline and threshold was not performed within a preset time period (50 ms). This time limit was long enough to allow for the kinematic variation of whisking reported in Figures 2C–E but short enough to discourage very slow whisking or lingering between baseline and threshold position. For trained mice, light OFF thus indicated a “ready signal,” which allowed them to initiate the trial in a self-determined fashion by whisking across the baseline. Once baseline and threshold were crossed sequentially, the trial was counted as successful, and the mouse immediately received a drop of water. In this case, the light stayed OFF for another 2 s to allow reward consumption. Immediately after this consumption period, the contingencies of licking/whisking and light were switched back to the ones shown in Figure 1B, and the next trial could be enabled and initiated by the mouse. If, in a trial attempt, the whisker crossed baseline but not the threshold line in the preset time, the light was set ON and the trial was aborted. In the first few sessions, baseline and threshold coincided and were located very close to the resting position, so the task was easy to accomplish even by chance, and the success rate was high. With successful learning of the task and increasing performance, the baseline was moved ca. 5 mm rostral to the resting position, and the dynamic relocation of the threshold was enabled. This measure required higher amplitude whisks as compared to the shaping period before and largely avoided unintentional baseline crossings (e.g., when the mouse took a break or disengaged from the task). In this study, only the sessions are reported, in which a well-trained animal worked on a baseline >5 mm rostral from rest and experienced automatic relocation of the threshold. The relocation was determined by the number of successes within the most recent five valid trials. The threshold moved in rostral direction by a margin of 0.93 mm, making the required whisking amplitude higher and the task harder, whenever the last five trials contained four successful trials. At this point, the 5-trial reward history was reset to zero. The paradigm ended when the mice were satiated and disengaged from the task. Whisker trajectories along with other behavioral parameters were recorded using Multi-Channel-Systems software (Reutlingen, Germany). Of note, two sessions/day were performed for all three animals, lasting each ∼25 min.

### Microstimulation and Lesions

A unilateral craniotomy was performed, under anesthesia with ketamine 100 mg/kg and xylazine 15 mg/kg (i.p.), to remove the skull overlying the motor cortex and to retract the dura. The location of the vibrissa representation of RW was mapped out by using intracortical microsimulation (duration 2 s, intensity 50 μA, bipolar cathodal pulse duration 200 μm; pulse rate 60 Hz; depth from pia 0.8–1 mm) to elicit rhythmic whisker movements (detected visually using the operation microscope at highest magnification). Specifically, the border to retraction-face area (RF, cf. Haiss and Schwarz, 2005) was sought and marked. A unilateral aspiration of the physiologically identified cortical tissue was performed with care being taken not to encroach the subcortical white matter and neighboring RF. Following completion of the surgery, the cavity was filled with Gelfoam along with a thin layer of dental cement, the skin was sutured to cover the exposed region. The postoperative care was performed as described earlier.

### Data Collection and Analysis

The real-time behavior was controlled using a MATLAB/Simulink real-time application (Natick, MA, United States), which ran at a sampling rate of 10 kHz. Behavioral parameters were recorded using Multi-Channel-Systems MCRack software (Reutlingen, Germany). Data analysis was carried out using custom-built MATLAB scripts.

The principal component analysis (PCA) was applied to quantify the change in whisker trajectories across thresholds. To this end, the baseline was subtracted from the whisking traces, and snippets (duration -10 to 50 ms with 0 = threshold crossing) were extracted. The whisking snippets, across all sessions and animals, were pooled together and served as the input to the PCA. From the trials applying the same threshold (of each session), the threshold mean was computed. The Euclidean distances between each of these threshold means within each session were computed. Statistical comparison between pre-lesion control and each group (2-week post-lesion and late post-lesion) was computed by the Wilcoxon tests. To visualize the difference in waveforms, the ensemble of whisks was projected to the area spanned by the first two Eigenvectors. The Euclidean distances between the center of mass obtained from whisks rendered with one threshold level were used as the quantification of the change of waveforms.

The change-point analysis was performed using a cumulative sum (CUSUM) algorithm (Taylor, 2000b). The algorithm first calculates the CUSUM series, the CUSUM of differences between signal (here, a trial series of maximal whisk amplitudes or velocities), and its mean. The absolute extreme of the CUSUM signals identifies either a mean-shift downward (maximum) or upward (minimum). Identified CUSUM extremes (=change points) were compared to 1,000 bootstrapped samples, which is generated by performing the identical analysis on a randomly permuted trial series. A CI was assigned to the found change point by finding its percentile in the bootstrapped ensemble. The initial input to the CUSUM analysis was the two trial series with constant thresholds before and after an increment trial. This eventually yielded the identification of a change point at level 1. The change points of the higher level (up to level 3) were also identified by dividing the signal into two parts (before and after the change point identified on the lower level) and repeating the procedure. We accepted all change points showing a CI > 0.7. Elevating the CI criterion to 0.95 yielded reduced numbers of change points, as expected. However, the ensembles of change points at both CI levels showed almost identical distribution across peri-increment trials. All peri-increment trial series tested for change points were checked for autoregression using the pattern test suggested by Taylor (2000a). None of the maximal amplitude and velocity series used for change point analysis (*n* = 93 each from pre-lesion sessions and 265 each from post-lesion sessions) were found to be significantly autoregressive (*p* < 0.05).

The effect size was defined as the area under the receiver operating characteristic curve (AUC). It is the probability of a binary classifier to correctly classify (using varying thresholds to strip off the observer bias) confronted with a data point randomly picked from the two distributions. Therefore, AUC = 0.5 signifies random performance while the AUC values of 0 and 1 signify perfect discrimination.

## Results

We trained and recorded the whisker motor behavior of three mice (#1–3), which were under water control and head-fixed, tracking one whisker (C1) on the left side of the snout. To define movement trajectory, start- and end-points were brought under experimental control using continuous reinforcement, i.e., the mouse determined the trial by enabling and then initializing the trial by its own volition. To enable the trial, whisker C1 had to be brought behind a baseline, i.e., a light cue indicated this state to the animal. To initialize the trial and gain a water reward, the whisker had to be swept from the baseline to a variable threshold within 50 ms. The movement was in air without touching external objects. The position of the whisker, enforced by a polyimide tube, was tracked by an optical device and sampled by a real-time controller, which decided online about the status of the house light. The house light was a cue for the mouse that signaled correct behavior when OFF and failure or discouraged behavior when ON (Figure 1A). The light was switched OFF when the mouse brought the whisker behind baseline into starting position, moved it across baseline and threshold within a set time limit, and during reward delivery and consumption. The light went ON when the whisker was held in front of the baseline when the mouse generated a premature lick (before water reward) or went beyond baseline but not beyond the threshold in a preset time (Figure 1B). To quantify whether mice are able to adapt their movements to changing demands, we introduced a procedure that increasingly required larger whisker protraction. This adaptive procedure begun in every session with the threshold being identical to the baseline. After the mouse reached 4 successful trials within a moving set of five, the threshold was slightly moved forward increasing the gap between baseline and threshold step by step (step size 0.93 mm). Whisker signal, baseline and threshold, licks, water delivery, and state of house light were monitored throughout the session. A successful whisker movement from behind the baseline across both baseline and threshold is shown in Figure 1C. All animals successfully learned the task in an initial training phase lasting for a minimum of 3 weeks with a minimum of one session on workdays. Once they performed continuously and successfully, generating hundreds of trials per session, they performed on 13 pre-lesion sessions (three in mouse #1, six in #2, and four in #3). These sessions encompass between 160 and 664 (median 253) trials, of which between 13 and 92 (median 55) were valid and led to a reward. The temporal progression of the threshold levels for these sessions is shown in Figure 1D. The maximum level ever reached was 13 (step-size 0.93 mm).

We next extracted successful whisks across baseline and threshold. Figure 2A shows the snippets of the whisker trace of one example pre-lesion session obtained from mouse #2 aligned to baseline crossing (left, at 15.81 mm) and threshold crossing (right, at 15.81–22.32 mm). The successful trials observed with each threshold level are plotted separately (t1–t8, from the initial threshold at baseline to the final and eighth level placed 6.51 mm further in front). Each threshold was mastered well by the mouse, responding with adequate whisking traces to yield rewards, indicating that its behavioral response dealt well with the requirement of this task. Importantly, the whisking traces reveal that the mouse dynamically adapted its whisk waveform to the actually valid threshold, rather than adjusting one large-amplitude waveform that would yield reward with all thresholds. The maximum whisk amplitudes (red circles) tended to be reached late with low thresholds and successively earlier with high thresholds. To quantify this change of whisk trajectories, we performed the PCA of the threshold-triggered whisking traces. This analysis would capture changes in all aspects of the whisk-waveforms. In Figure 2B, we plotted the 2D projection spanned by the first two principal components which revealed a gradual shift of the shape of whisks (empty circles) and their center of mass (filled circles) for successive thresholds (colors). To reveal the kinematic outline of adaptive whisking, we plotted the maximum value of position, velocity, and acceleration of single whisks in Figures 2C–E. The maximum (most rostral) position was found to be systematically adjusted to the threshold levels reaching on average 2.9 mm beyond the threshold. It was interesting to note that velocity was modulated in conjunction with reach, yielding similar Pearson’s correlation strength (position: *r* = 0.65, velocity: *r* = 0.56, *p* < 0.05), but not acceleration, which showed minimal and non-significant correlation with threshold levels (*r* = 0.03, *p* > 0.05). This finding demonstrates that the example mouse responded to the increasing levels of thresholds and adapted its whisks to yield a position that would just exceed the threshold. We then investigated how these adaptations come about during the behavioral session. We found that mice use the dependence of threshold increments on performance. With four rewards gained in a group of five trials, the threshold would shift forward. Therefore, we expected that the probability of gaining a reward should be high five trials before a threshold increment. This expectation was met in the population data from all mice (Figure 2F, green). Interestingly, the mice clearly expected higher thresholds after a series of high reward gains, because there was no breakdown in reward probability immediately after threshold increment. Mice adjusted their whisking amplitudes and velocities immediately to higher levels as in the period before (Figure 2F, red and blue). We confirmed this finding by calculating change points, i.e., a statistical method to identify shifts of the mean (refer to the “Materials and Methods” section), in a series of maximal amplitude and maximal velocity across trials. Across the pre-lesion sessions of the three mice, we searched for change points in the vicinity of trials with threshold increments (±20 trials). From 141 change points detected in the maximal amplitude series (52 “shift-down,” 89 “shift-up”) and 122 change points detected in the maximal velocity series (46 “shift-down,” 76 “shift-up”), we identified an elevated probability of upward shifting change points in maximal amplitudes/velocities at about five trials before an increment and a counteracting shift downward immediately after the threshold increment (Figure 2F, magenta and violet). The downward shift, however, was smaller in amplitude, as mentioned earlier so that the maximal amplitude and velocity reached the new threshold level (Figure 2F red and blue, compared with the example session shown in panels C,D).

**FIGURE 3:**
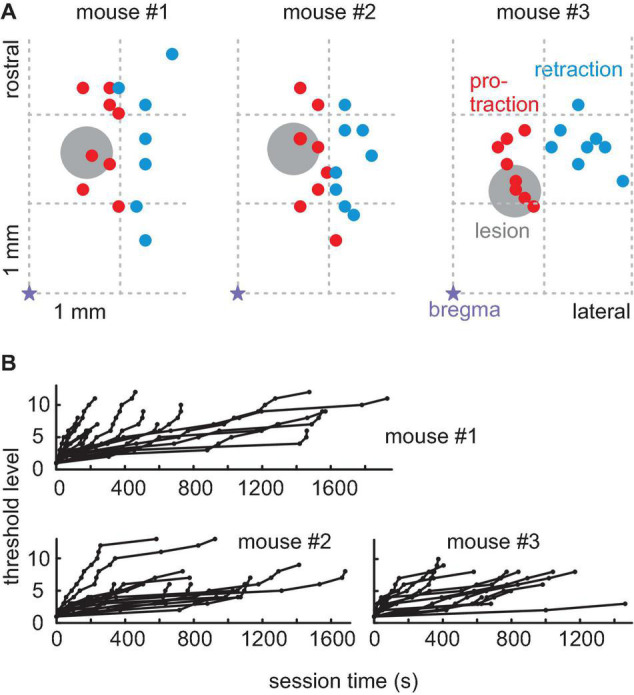
Rhythmic whisking (RW) lesions. **(A)** The location of minimal lesions within the functional map of the right hemisphere whisker motor cortex for three mice. The violet star is bregma; right is lateral; up is rostral; and the grid indicates the location in anterior-lateral coordinates. The colored dots are the locations of successful microstimulation evoking protraction (red) and retraction (blue whisker movements). Lesions (diameter about 0.5 mm, gray circle) were done with a micro-suction pipette. They were located within RW (red) but in the vicinity of RF (blue). **(B)** Progression of threshold levels in all post-lesion sessions in all three mice (conventions as in Figure 1D).

**FIGURE 4:**
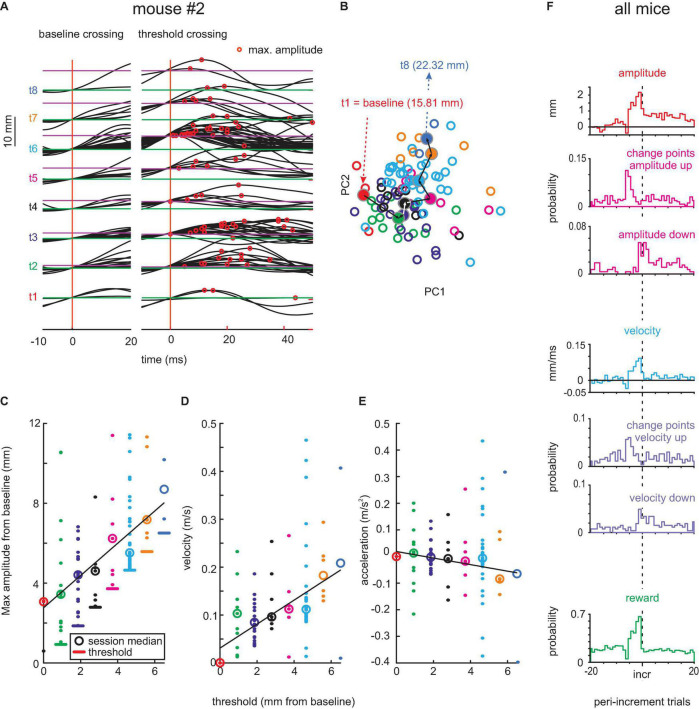
Adaptive whisking up to 12 days after contralateral RW lesion. **(A–E)** Example session. **(F)** Population data. Same conventions as in Figure 2.

**FIGURE 5:**
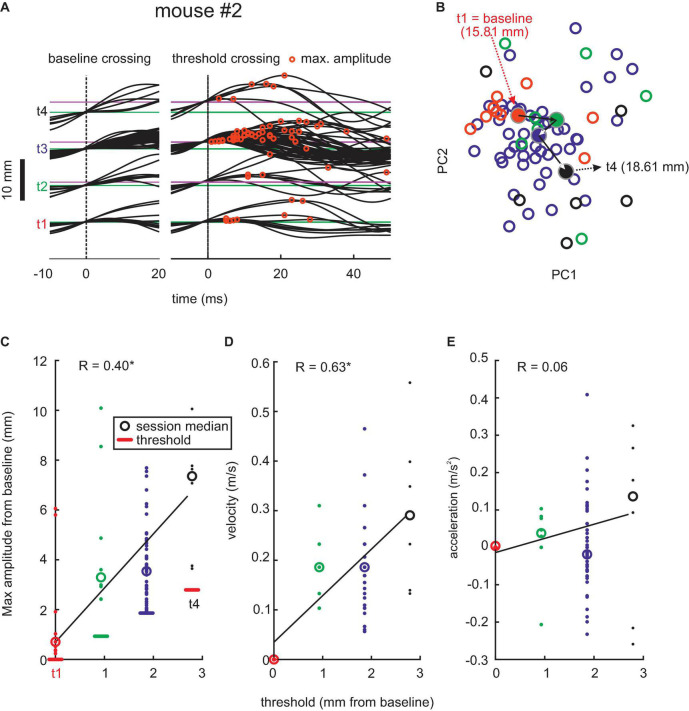
Adaptive whisking later than 12 days after contralateral RW lesion. Example session. **(A–E)** Same conventions as in Figures 2, 4.

Next, the primary motor cortex area RW, which is the area responsible for oscillatory whisking and protraction (Haiss and Schwarz, 2005; Cramer and Keller, 2006; Ferezou et al., 2007), was lesioned in all three mice (Figure 3A). To introduce minimum effects, we used intracortical microstimulation pulse bursts in the motor cortex and mapped evoked protraction vs. retraction movements (Haiss and Schwarz, 2005). A circular area of about 0.5 mm diameter, located well in the RW range, was removed by suction. The mice were then reintroduced to the task between days 6 and 11 after the lesion. We recorded a total of 56 sessions (mouse #1: 18 sessions; mouse #2: 20 sessions; mouse #3: 15 sessions) with a median of 260 trials (range 38–1,083) and a median of 44 successful trials (range 4–146) of which a median of 29.5 successful trials (range 5–90) was before post-lesion day 12 and a median of 48.5 successful trials (range 4–146) was after post-lesion day 12. The shifts in threshold levels obtained in the post-lesion sessions are shown in Figure 3B. The last behavioral recordings were made at 39 (animal #2) and 46 (animals #1 and #3) days after lesion, respectively. The effects of RW lesions depended on the time after lesion. Until post-lesion day 12, there was a minimal effect on task performance. The lesion, however, was effective as it compromised whisker movements later on. We, therefore, divided the post-lesion data into two periods, namely, one until post-lesion day 12 (“early post-lesion,” Figure 4) and the other later than post-lesion day 12 (“late post-lesion,” Figure 5).

Early post-lesion behavior was virtually unchanged as compared to pre-lesion data. Without contralateral RW, mouse #2 was able to move its whisker and readily adapted it to the threshold shifting away from baseline (Figure 4A). The progression of whisk waveforms as demonstrated by PCA showed a similar systematic progression as in the pre-lesion period. Also, the adaptation of kinematic parameters showed an unchanged profile, yielding clear adaptation of whisk amplitude and velocity (position: *r* = 0.58, *p* < 0.05; velocity: *r* = 0.37, *p* > 0.05) but not acceleration (*r* = -0.04, *p* > 0.05; Figures 4C–E). Across the three mice, 408 change points for maximal amplitude series (“shift-down” 149; “shift-up” 259) and 378 change points for maximal velocity series (“shift-down” 163; “shift-up” 215) were found (Figure 4F). The strategy to adapt whisking parameters, such as amplitude and velocity, and their dynamics as indicated by change points was unchanged as compared to the pre-lesion data.

Lesions in RW, however, led to clear changes in motor behavior starting about 12 days after the lesion (Figure 5). All three lesioned mice started to become unable to generate whisks of higher amplitude at that time and, therefore, had difficulties to reach higher thresholds in the task. Fewer successful threshold-surpassing movements per session were observed. The whisking trajectories were more similar, and PCA did not show clusters that migrated as much as in pre-lesion and early post-lesion sessions. Despite these deficiencies, there was a significant increase in amplitudes and peak velocities with increasing threshold levels, which as in the pre-lesion data gave significant correlation coefficients (position: *r* = 0.40, *p* < 0.05; velocity: *r* = 0.63, *p* > 0.05). Again, similarly in control sessions, acceleration was scarcely changed with increasing threshold levels. The late onset of these effects and the survival of certain features of adaptability (amplitude and velocity adjustments) exclude an “instructive role” of the minimal RW lesions for the observed whisking behavior (refer to the “Discussion” section).

**FIGURE 6:**
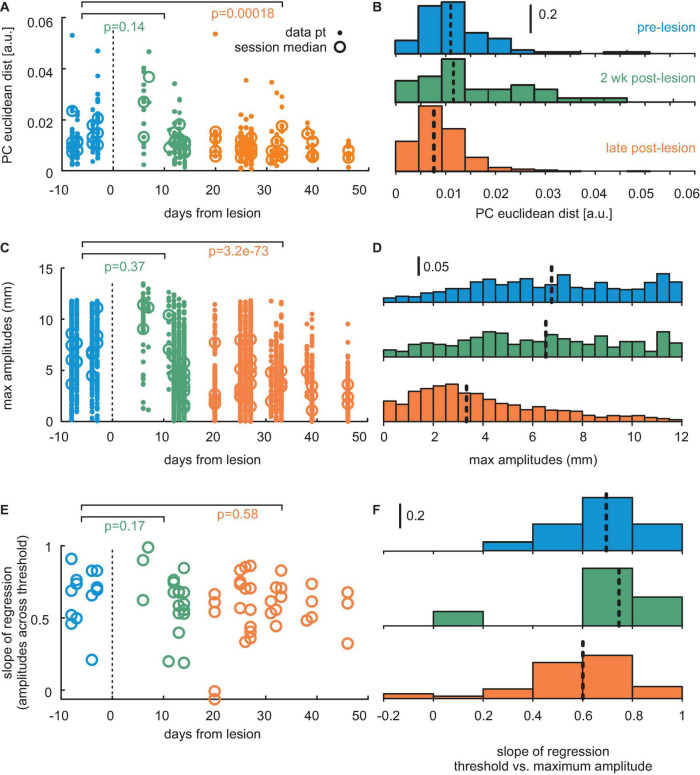
Population data from three mice. **(A)** Distance of center of mass (PC1 vs. PC2) between whisker trajectories generated with different thresholds. The *p*-values are denoted. Dots: single sessions. Circles: session means. Blue: pre-lesion data. Green: Data obtained up to 12 days post-lesion. Orange: Post-lesion data obtained later than 12 days after lesion. **(B)** Same data as the dots in panel **(A)**, now depicted as a histogram. Colors match. Broken lines: sample median. **(C)** Maximum positions of whisks. Conventions as in panel **(A)**. **(D)** Same data as in panel **(C)**. Conventions as in panel **(B)**. **(E)** Adaptation expressed as the slope of regression line obtained with threshold and maximum position (cf. panels C in Figures 2**,** 4**,** 5). Conventions as in panels **(A,C)**. **(F)** Same data as in panel **(E)**. Conventions as in panels **(B,D)**.

Figure 6 shows population data from all three mice. We observed a significant decline in the adaptation of whisks to task demand in the late post-lesion period, indicated by a reduction of migration distance of the center of mass of whisk shape in PCA vector space (*p* < 0.001, *n* = 86 pre-lesion and 196 late post-lesion PCA vectors, Wilcoxon signed-rank test). Figures 6A,B shows results from the PCA using all whisking trajectories across trails, sessions, and mice as input. The effect is mediated by the decreased ability to generate large-amplitude/high-speed whisks late in the post-lesion phase (*p* < 0.001, *n* = 700 pre-lesion and 2,392 late post-lesion whisks, Wilcoxon signed-rank test, Figures 6C,D). The role of whisk amplitudes for the adaptation to the task is expressed in the high correlation of maximal amplitude generated with the increasing threshold (Figure 6E). It is, however, noteworthy that even in the late post-lesion period, a good deal of the ability to adapt to stimulus demands survived (cf. Figure 5). On average, the three mice showed a correlation coefficient of maximum amplitude of whisks with threshold level of 0.69 for pre-lesion sessions; 0.68 for early post-lesion sessions; and 0.6 for late post-lesion sessions (Figures 6E,F).

## Discussion

Mice are able to adapt their whisker reach trajectories according to the availability of reward. With reward-yielding targets moving to increasingly distant locations, they respond by adapting their whisker reach in a goal-oriented way. Mice use reward history to adapt their kinematic whisker pattern, i.e., increasing maximal amplitudes and velocities with stable maximal accelerations. Without significant load, this kinematic layout is compatible with the notion that to generate farther reaches, the applied muscle force is unchanged but kept for a longer time period. Thus, the performance of the task tests memory and cognitive functions as well as movement generation. Structural lesions within contralateral RW of about 0.5 mm diameter did affect neither adaptation strategies based on evaluating reward history nor changes in whisking trajectory. However, the paradigm helped to reveal specific secondary effects at 12 days post-lesion. Contralateral RW lesions led to the inability of the animals to reach high-amplitude whisks, while largely keeping their ability to systematically adapt whisking amplitudes.

### How Versatile Is Whisking?

Our results show that kinematic whisking parameters are changed in a systematic and goal-oriented way. While the adaptability of whisking is commonly taken for granted, quantitative evidence in the literature for it has been scant so far. On the biomechanical level, there is evidence that the discriminability of textures can be optimized by whisking velocity (Oladazimi et al., 2018); however, whether animals use this opportunity is uncertain. One study suggested that the main frequency and set points may be specifically set for object discrimination (Carvell and Simons, 1995). However, the same publication and another one (von Heimendahl et al., 2007) also showed that the most important parameter for discrimination success seems to be the time of touch and/or the number of palpation strokes. Furthermore, it is not finally understood whether whisking is systematically applied or withheld to optimize discrimination. For instance, observations from different object location tasks do not coincide in their conclusions whether whisking is critical for task performance (Krupa et al., 2001; Knutsen et al., 2006).

With respect to the question of versatility, it is important to point out that whisking is an active scanning system, such as echo- or electrolocation. Such systems deploy energy into the environment, for the respective sensory mechanisms to receive and make use of the reflections evoked by these deployments. It is a hallmark of active scanning systems that energy deployments are highly structured, similar to spectral features of bat calls (Schnitzler and Kalko, 2001) or waveforms of electric discharges in fish (Caputi, 2021). Whisking, using stereotyped, highly rhythmic whisker movements, is no exception (Welker, 1964; Bermejo et al., 1998). Nevertheless, whisking is a major sense in nocturnal rodents, and general behavioral observation is strongly suggestive of varied functional purposes. Whisking-related behaviors are known to range from sensorimotor servo-functions, object touch and discrimination, navigation, attacking, swimming, social touch, and others (Gregoire and Smith, 1975; Ahl, 1982; Carvell and Simons, 1995; Brecht et al., 1997; Towal and Hartmann, 2006; Mitchinson et al., 2007; Bobrov et al., 2014).

In this study, we quantitatively studied the adaptability of whisking. Our results have shown beyond doubt that mice adapt their whisker reaches toward dynamically relocated targets. The task, in many aspects, is comparable to reaching used in the studies of hand/arm movements in humans and monkeys (Georgopoulos et al., 1982; Shadmehr and Mussa-Ivaldi, 1994). Similar to arm-reaching tasks in primates, our task is likely amenable to be used to engage different motor learning systems. With our present implementation, we were able to show that reach adaptation was guided without sensory feedback about the target. Mice instead used reward prediction errors to adapt whisking (Izawa and Shadmehr, 2011). The dominant strategy applied by the mice was to use the information offered by reward history about upcoming threshold increments to adjust their whisking kinematics. This clearly is a higher-order anticipatory strategy that must have involved the short- and/or long-term memory as well as computing executive signals. In future applications, the task may be used to present target locations that cannot be anticipated. It lends itself also for studies of motor learning using reward- or sensory-prediction errors, e.g., by decoupling threshold increments from reward history, or allowing sensory feedback, e.g., by using tangible targets.

### Capturing the Effect of Minimal Lesions in Rhythmic Whisking

Previous whisking tasks (Haiss and Schwarz, 2005) are insensitive to the difference in effects on the computation of the trajectory vs. higher-order planning. We used miniature lesions in the RW subarea to test whether our test is able to illuminate such a difference. These lesions did not affect whisking directly after lesion, as in all animals, we obtained post-lesion sessions showing unaffected whisking trajectories and adaptation. Thus, the small lesions of RW are non-critical to compute trajectories as well as adaptation. These results were expected from the previous observation that even large wM1 lesions led to relatively subtle effects on spontaneous whisking (Gao et al., 2003; Ebbesen et al., 2017). However, our task successfully differentiated secondary effects on trajectories that left reach adaptation intact. With this result, we first demonstrated that the novel task is suitable for the differentiation of detailed motor roles. Second, the result suggests that trajectory computation and adaptive whisking are the functions that are disjunct in terms of behavior and in terms of neuronal systems underlying them. Our present data do not detail where, in the mouse motor system, these neuronal functions are computed. Small RW lesions affect trajectories only but leave reach adaptation intact. Based on the present data, we cannot exclude the possibility that the remaining RW or RF subarea of wM1 is involved. However, previous findings that large lesions encompassing the entire wM1 did not abolish whisking entirely but rather subtly reduce whisker trajectories predominantly on the contralateral side (Gao et al., 2003; Ebbesen et al., 2017). It is further a well-corroborated observation that the vast majority of unit recordings in wM1 are only loosely related to the details of the whisking trajectory (Hill et al., 2011; Gerdjikov et al., 2013; Ebbesen et al., 2017) and that the time series and frequencies of stimulus command only have a minor effect on whisker trajectories (Haiss and Schwarz, 2005). Taken together, these known characteristics are consistent with a role for wM1 on a higher level of whisking control, perhaps adapting whisker movements to goals, as requested here. Whisker-M1 microstimulation has revealed functional modules (Schwarz and Chakrabarti, 2015), with one functional subarea evoking retraction movement (RF) and the other (RW) giving rise to protraction and oscillatory movements (Haiss and Schwarz, 2005; Ferezou et al., 2007; but refer to Auffret et al., 2018). In this study, we focused on RW, which is the most relevant of the two, as its stimulation in awake rodents evokes protraction and rhythmic movements, similar to the movements we trained our mice to perform. We chose to perform structural lesions, as the observation of whisking adaptation for an extended period after the lesion would give us insight into whether RW is “permissive” (which can disturb the behavior but *does not implement* relevant computations required to enact the behavior) or “instructive” (which does the relevant computation) for whisking (Otchy et al., 2015; Stüttgen and Schwarz, 2018). Our results are clear-cut, and the whisking adaptation is unimpaired in a period of 12 days after small RW lesions. That is, either intact parts of wM1 are able to uphold adaptation, or wM1 is not involved in adaptation at all. The acute pharmacological blockade of wM1 has been reported to have more detrimental effects on whisking (Huber et al., 2012; Ebbesen et al., 2017; cf. for a related digression of results with lesion vs. acute blockade in the paw motor system: Guo et al., 2015; Kawai et al., 2015). In view of the comparably mild effects of permanent lesions seen here and previously (Gao et al., 2003), we argued that reported blockade of wM1 may well be based on comparably short-lasting homeostasis effects, i.e., being “permissive” (Stüttgen and Schwarz, 2018). In our view, the effects of transient blockade of wM1 must be interpreted with caution in terms of “instructive” roles for the generation of detailed whisking trajectories and reach adaptation.

Despite the lack of immediate effects on whisking generation and adaptation, our results, at least indirectly, corroborate that even the minimal lesions of RW performed here, lead to rather strong effects on the control of whisking. After a post-lesion period of 12 days, whisking trajectories deteriorated in all three lesioned mice (while reach adaptation, surprisingly, was kept intact). The deficiency manifested itself in the inability to reach distant targets, such that in one mouse, the starting point of the operand whisk had to be moved backward for the animal to be able to do the task. The other two mice never reached the distant target levels anymore, as performed at ease in the pre-lesion and the immediate post-lesion period. In view of the small size of the lesions, this result is surprising and suggestive for an indirect role of RW to generate whisker trajectories. The effects are consistent with the processes of plasticity and/or reorganization in the motor system at large triggered by the lesions of RW. The consistency of these results in terms of temporal progression and specific elements of affected behavior do not argue that they were due to any unforeseen side effects of lesioning but that they were due to systematic processes following the lesion. One such possible mechanism consists in degenerating RW terminals, either at a cortical site or at the synaptic input level of the central pattern generator (CPG), causing a functional disbalance and/or sprouting of other terminals to fill the gaps.

In conclusion, we have established a task that allowed us to unequivocally state that whisking is not stereotyped but can be adapted to goals in systematic ways. Small lesions in contralateral RW do not affect such adaptation, arguing against the idea that trajectories are computed inside RW. However, the late effect on whisker trajectories suggests the intricate involvement of RW in the organization of whisking trajectories.

## Author’s Note

The current affiliation of SC is the MathWorks, Germany. JN moved to the Brain Research Institute University of Zurich, Switzerland.

## Data Availability Statement

The raw data supporting the conclusions of this article will be made available by the authors, without undue reservation.

## Ethics Statement

The animal study was reviewed and approved by Regierungspräsidium Tübingen.

## Author Contributions

SC designed and conducted the experiments, analyzed the data, and wrote the manuscript. JN conducted the experiments and analyzed the data. CS designed the experiments, supervised the experimental work, and wrote the manuscript. All authors contributed to the article and approved the submitted version.

## Conflict of Interest

The authors declare that the research was conducted in the absence of any commercial or financial relationships that could be construed as a potential conflict of interest.

## Publisher’s Note

All claims expressed in this article are solely those of the authors and do not necessarily represent those of their affiliated organizations, or those of the publisher, the editors and the reviewers. Any product that may be evaluated in this article, or claim that may be made by its manufacturer, is not guaranteed or endorsed by the publisher.
